# Speaking the same anatomical language: standardizing laryngeal nerve terminology matters in anatomy and medicine

**DOI:** 10.1016/j.bjorl.2026.101884

**Published:** 2026-10-02

**Authors:** Jaiurte Gomes Martins da Silva, Eduarda Correia Moretti, Philippe Oliveira Lima, Darlisson Rodrigues Duarte, Abrahao Verçosa Amorim Filho, Anderson Fernandes Silva, Glícia Maria de Oliveira, Jodonai Barbosa da Silva, Ivson Bezerra da Silva, Mariana de Carvalho Leal Gouveia

**Affiliations:** a Universidade Federal de Alagoas (UFAL), Complexo de Ciências Médicas e Enfermagem (CCME), Arapiraca, AL, Brazil; b Universidade Federal de Alagoas (UFAL), Instituto de Ciências Biológicas (ICBS), Maceió, AL, Brazil; c Universidade Federal de Alagoas (UFAL), Faculdade de Medicina, Maceió, AL, Brazil; d Universidade Federal de Pernambuco (UFPE), Centro Acadêmico de Vitória (CAV), Vitória de Santo Antão, PE, Brazil; e Universidade Federal da Paraíba (UFPB), Centro de Ciências da Saúde (CCS), João Pessoa, PB, Brazil; f Universidade Federal de Pernambuco (UFPE), Centro de Ciências Médicas, Recife, PE, Brazil

**Keywords:** Recurrent laryngeal nerve, Anatomy, Terminology, Clinical medicine

## Abstract

**Objectives:**

This study aimed to identify the most appropriate terminology for the nerve commonly referred to as the “recurrent laryngeal nerve” or “inferior laryngeal nerve”, based on official anatomical nomenclatures. Additionally, it sought to analyze the frequency of use of these terms in scientific literature and provide a detailed anatomical description of the nerve to promote terminological accuracy in academic and clinical settings.

**Methods:**

A descriptive analysis was conducted in three phases. First, a literature search was performed in MEDLINE/PubMed, Embase, LILACS, SciELO, Cochrane Library, and Google Scholar, alongside a review of major anatomical textbooks, to quantify the usage of both terms. Second, the*Terminologia Anatomica* (TA) and *Terminologia Neuroanatomica* (TNA) were consulted to identify the officially sanctioned term. Third, a comprehensive description of the nerve's anatomy, function, and clinical significance was compiled from authoritative sources.

**Results:**

The term “recurrent laryngeal nerve” was overwhelmingly predominant in all searched scientific databases and modern anatomical textbooks. For instance, MEDLINE/PubMed yielded 1,828 articles using this term in the title, compared to only 111 for “inferior laryngeal nerve”. Both the TA and TNA exclusively recognize “N. laryngeus recurrens”, translated as “recurrent laryngeal nerve”, as the official designation. The term “inferior laryngeal” in current nomenclatures is reserved for vascular structures, namely the inferior laryngeal artery and vein.

**Conclusion:**

The correct and internationally accepted term is “recurrent laryngeal nerve”. The consistent use of standardized terminology is crucial for precise communication in education, clinical practice, and research. A thorough understanding of the recurrent laryngeal nerve's anatomical course and variations is essential for minimizing the risk of iatrogenic injury during surgical procedures in the neck, particularly thyroid and parathyroid surgeries.

## Highlights

• “Recurrent laryngeal nerve” is the correct and most used anatomical term.
• Official anatomical terminologies do not recognize “inferior laryngeal nerve”.
• Terminological precision is critical for safe thyroid and parathyroid surgery.
• Standardized nomenclature helps prevent iatrogenic recurrent laryngeal nerve.

## Introduction

A major challenge in education and health sciences is standardizing terminology. The inconsistent use of anatomical terms complicates student learning and compromises clinical reasoning, communication, and treatment planning.^1^ Although the *Terminologia Anatomica* (TA) was published to reduce discrepancies between traditional terms and eponyms,^2^ terminological inconsistency persists.^3^ Various studies have sought to align anatomical nomenclature with clinical applicability^4^ or promote corrections and expansions,^5^ leading the FCAT to release a provisional terminology version in 1998.^6^

The nervous system is particularly affected,^7^ which prompted the introduction of the *Terminologia Neuroanatomica* (TNA), a distinct set of terms.^8^ These spurred efforts to standardize nomenclature for cerebral gyri, sulci,^9^ and thalamic structures,^10^ informed by neuroimaging. The lack of standardization undermines both academic and clinical contexts: it complicates teaching and learning, and may compromise diagnostic accuracy and interdisciplinary communication.^11^^,^^12^

The objective of this study is to determine the most appropriate designation for the recurrent laryngeal nerve, also referred to as the inferior laryngeal nerve, based on the TA and TNA.^8^^,^^13^ Additionally, this study aims to analyze the use of these terms in indexed scientific publications and to describe the nerve's anatomical characteristics, thereby enhancing precision and clarity in literature and clinical practice.

## Methods

This study provides a descriptive analysis of the anatomy and terminological usage of the expressions “recurrent laryngeal nerve” and “inferior laryngeal nerve”. Conducted in July 2025 with the collaboration of anatomy professors from several Brazilian federal universities, the research was organized into three phases, as outlined below.

### Phase I: Terminological usage in scientific literature and textbooks

The first stage examined scientific databases and anatomical textbooks to assess the frequency of use of the terms “recurrent laryngeal nerve” and “inferior laryngeal nerve” in article titles and textbook content. On July 28, 2025, the following databases were searched: MEDLINE/PubMed, Embase, LILACS, SciELO, Cochrane Library, and Google Scholar. Searches were conducted using the exact phrases above, limited to occurrences in article titles, with no restrictions on language or publication date. The search strings were “recurrent laryngeal nerve”[ti] and “inferior laryngeal nerve”[ti] for MEDLINE/PubMed, with equivalent title-field syntax adapted for each database. Restricting the search to article titles was a deliberate methodological choice, as titles most directly reflect the author’s explicit and preferential terminology for the subject.

For the textbook analysis, key references were reviewed. Relevant sections concerning the larynx or the nerve were identified through the table of contents, and the content was then examined in detail to determine which term was consistently used in each source.

### Phase II: Identification of standardized terminology

The second stage sought to identify the officially recognized terminology in international anatomical nomenclature and determine which designation is most appropriate for scientific use. To this end, the most recent versions of anatomical terminology were consulted, including the Brazilian printed edition, the international online version, and the TNA.^8^

The Brazilian edition, translated and published in 2001 by Manole, was prepared under the coordination of the Anatomical Terminology Commission of the Brazilian Society of Anatomy, with support from the Federative Committee on Anatomical Terminology (FCAT). The electronic version, available since 2010, is hosted on the official website of the International Federation of Associations of Anatomists (IFAA) (www.unifr.ch/ifaa). During this stage, a targeted search was also conducted for all anatomical structures containing the expression “inferior laryngeal” in the TA.

### Phase III: Anatomical and clinical description of the nerve

The final stage involved compiling a detailed description of the anatomical course, functional role, and clinical relevance of the nerve. This was accomplished through a comprehensive review of authoritative anatomy textbooks and peer-reviewed articles, with the aim of supporting students, educators, researchers, and clinicians who rely on precise and standardized terminology.

## Results

### Phase I: Database and textbook searches

The MEDLINE/PubMed search retrieved 1,828 articles with the term “recurrent laryngeal nerve” in the title, 439 of which were published in the past five years. In contrast, the term “inferior laryngeal nerve” appeared in 111 titles, with only 10 published during the same period. In Embase, 2,119 records contained “recurrent laryngeal nerve” in the title (500 within the last five years), while 126 included “inferior laryngeal nerve” (13 within the last five years).

In the LILACS database, 18-articles were identified with “recurrent laryngeal nerve” in the title (3 from the past five years), compared with 9-articles using “inferior laryngeal nerve”, none published in the past five years. The SciELO search yielded 13-articles with “recurrent laryngeal nerve” in the title and 6 with “inferior laryngeal nerve”.

In the Cochrane Library, “recurrent laryngeal nerve” appeared in 1 systematic review and 81 clinical trials, whereas “inferior laryngeal nerve” was not found in systematic reviews and appeared in only 3 clinical trials. Searches via Google Scholar identified 3,420 documents with “recurrent laryngeal nerve” in the title, 770 of which were published since 2020. By comparison, “inferior laryngeal nerve” appeared in 214 documents, 22 published since 2020.

Analysis of anatomical textbooks revealed that “recurrent laryngeal nerve” predominated in widely used references, including those by Cunningham (1949), Lockhart, Hamilton, and Fyfe (1965), Aprile, Figún, and Garino (1967), Hamilton (1982), and Williams et al. (1995).^14^, ^15^, ^16^, ^17^, ^18^ Conversely, “inferior laryngeal nerve” appeared mainly in older works, such as Meckel et al. (1826), Sappey (1882), and Testut and Latarjet (1944).^19^, ^20^, ^21^ Notably, although these earlier texts introduced the structure as the inferior laryngeal nerve, they consistently referred to it as the recurrent laryngeal nerve throughout the description.

Two anatomy books ‒ Fusari (1913) and Chiarugi (1936) ‒ exclusively used “inferior laryngeal nerve” and made no reference to the term “recurrent” in their descriptions.^22^^,^^23^

Analysis of a recent major anatomical textbook further supports the continued predominance of “recurrent laryngeal nerve”. In the 9th edition of Moore ‒ Clinically Oriented Anatomy (2024), Dalley and Agur consistently use “recurrent laryngeal nerve” in their description of laryngeal innervation, with no use of “inferior laryngeal nerve” as a synonym.^24^ This finding reinforces the contemporary validity of “recurrent laryngeal nerve” as the standard designation in major anatomical references.

### Phase II: Consultation of international anatomical terminology

Both the Brazilian^13^ and online versions of the TA are organized into general sections and specific subsections corresponding to each body system. Accordingly, the section devoted to nervous system structures was consulted. In both versions, the content is presented in a three-column format: the first column provides the identification number of the structure, the second lists the official Latin term, and the third presents the equivalent term in the language of the consulted version (Portuguese or English).

In the Brazilian edition,^13^ terms related to the vagus nerve are located on pages 164–165. As the recurrent laryngeal nerve is a branch of the vagus nerve, it is listed on page 165. There, the recurrent laryngeal nerve is assigned the identification number A14.2.01.166, and its official Latin designation is “N. laryngeus recurrens” (Fig. 1).

**Fig. 1:**
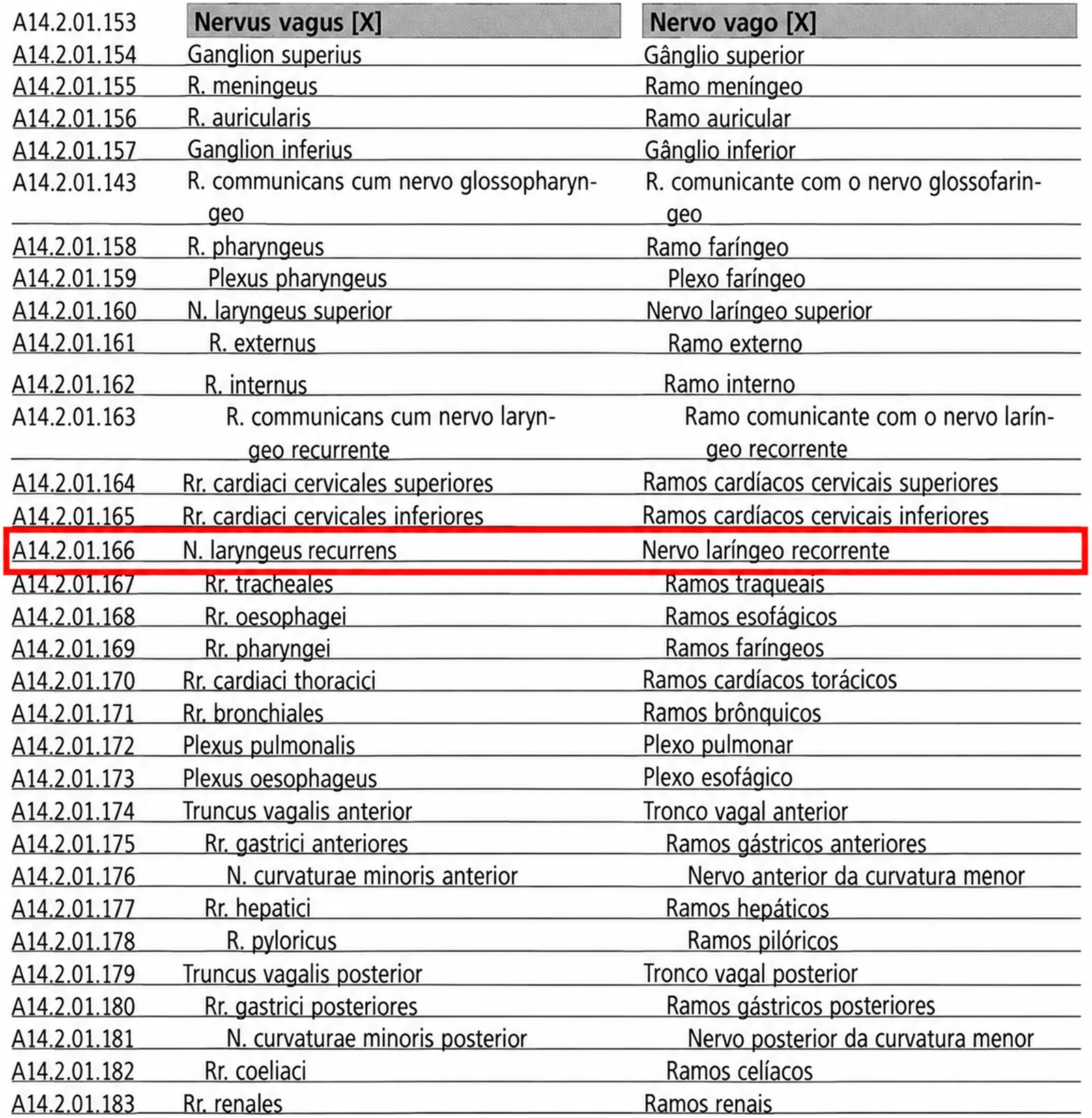
The anatomical terminology for the recurrent laryngeal nerve. Excerpt from the Brazilian edition of the *Terminologia Anatomica* (pages 164–165). The image displays the official identification number, the Latin designation, and the corresponding Portuguese term for the nerve.

To confirm these findings, the online version of the TA was consulted using the identification number corresponding to the recurrent laryngeal nerve. The same Latin term, “N. laryngeus recurrens”, was identified, and the equivalent English term was listed as “recurrent laryngeal nerve”, with no mention of the word “inferior” (Fig. 2).

**Fig. 2:**
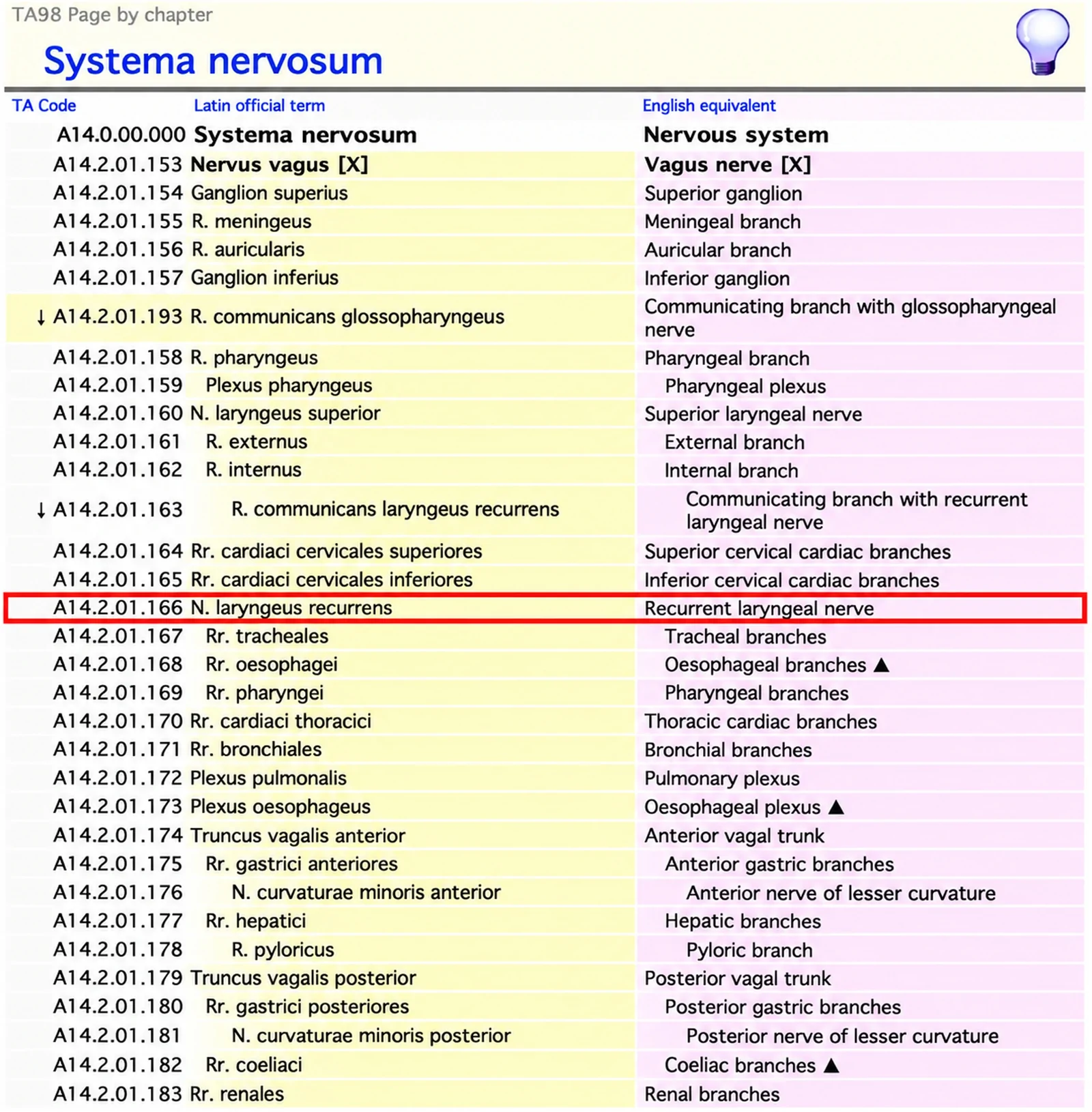
The entry for the recurrent laryngeal nerve in the online *Terminologia Anatomica* (TA) (www.unifr.ch/ifaa). The page displays the identification number (TA code), the official Latin designation, and the corresponding English term. TA, *Terminologia Anatomica*.

A targeted search of the online TA for anatomical structures containing the words “inferior laryngeal” identified only two entries: the inferior laryngeal artery and the inferior laryngeal vein, both of which are vascular structures (Fig. 3).

**Fig. 3:**
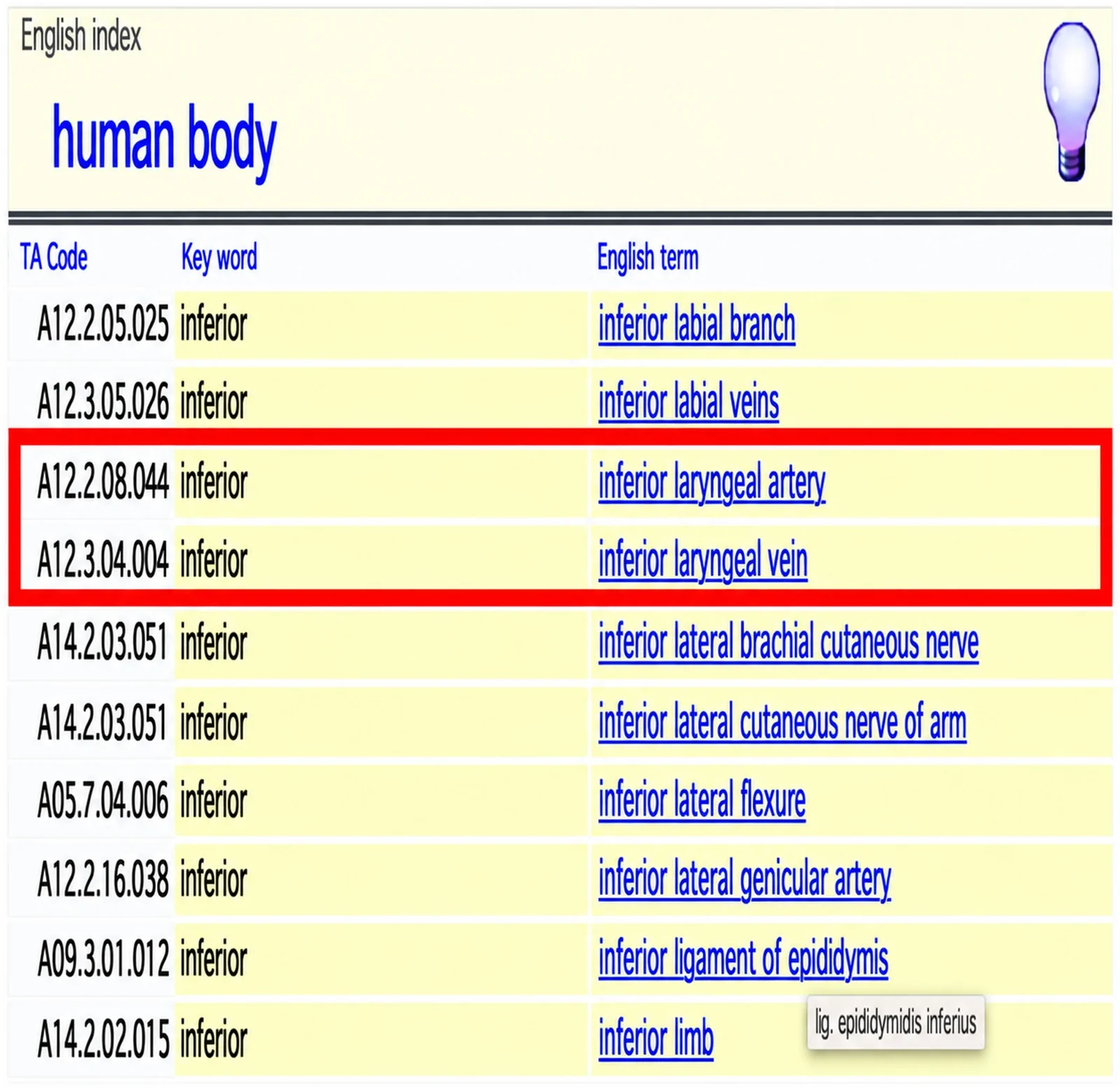
Online terminology for structures named “inferior laryngeal”. Screenshot from the online version of the *Terminologia Anatomica* (TA), available at www.unifr.ch/ifaa. The image displays the entries for anatomical structures containing “inferior laryngeal” in their names, showing the identification code (TA code), official Latin designation, and corresponding English term for each.

Finally, the TNA^8^ was also consulted, which confirmed “recurrent laryngeal nerve” as the appropriate designation (Fig. 4).

**Fig. 4:**
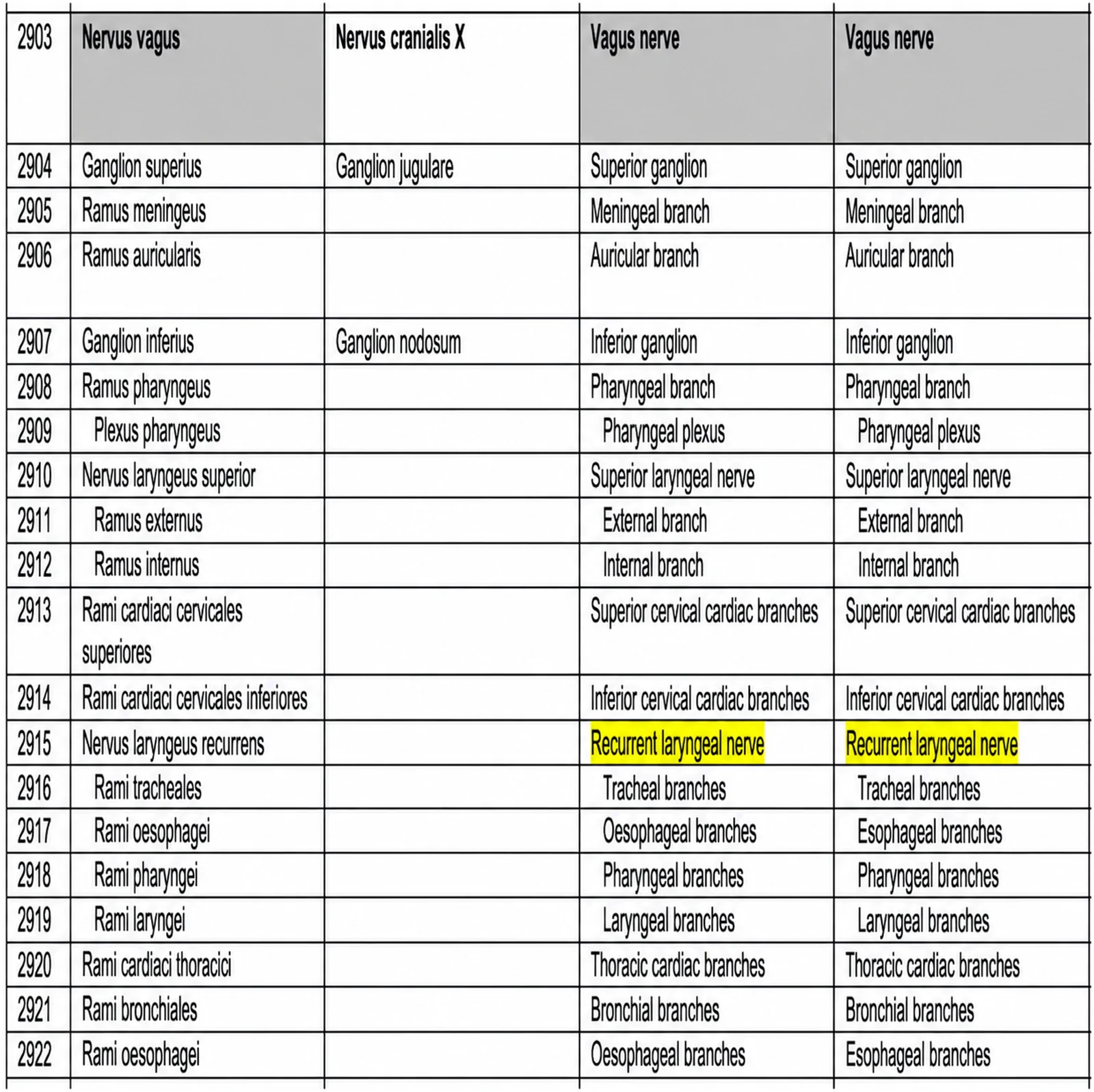
Neuroanatomical terminology for the recurrent laryngeal nerve. Excerpt from the *Terminologia Neuroanatomica* (page 100). The image displays the identification number, the official Latin designation, and the corresponding English term for this nerve.

### Phase III: Recurrent laryngeal nerve anatomy

The Recurrent Laryngeal Nerves (RLN) emerge from the vagus nerves (10th cranial nerve), in both sides (left and right), in the inferior part of the neck. The nerves have essentially the same distribution bilaterally; however, they loop around different structures and at different levels comparing the two antimers.^24^ The RLN receives this name because the term recurrent from latin: re (means back) and currere (means to run), and it indicates the nerve runs in the opposite direction to the vagus nerve from which they branch. The vagus nerves run down into the thorax, and the recurrent laryngeal nerves run up to the larynx.^25^

The RLN was described by Galeno of Pergamo (130‒210 d.C.), and this nerve carries motor, sensory and parasympathetic fibers to the larynx.^26^ As previously showed, the RLN runs a different course on each side: on the right antimer, this nerve loops below of the first part of the subclavian artery, then ascends toward the transesophageal groove before entering the larynx. On the left antimer, the RLN comes from the vagus nerve at the aortic arch and returns into the neck within the transoesophageal groove.^27^ In both sides, the RLN supplying trachea and esophagus and all intrinsic muscles of the larynx (thyroarytenoid, posterior cricoarytenoid, lateral cricoarytenoid, vocalis, transverse and oblique arytenoids), except the cricothyroid^24^ (Fig. 5).

**Fig. 5:**
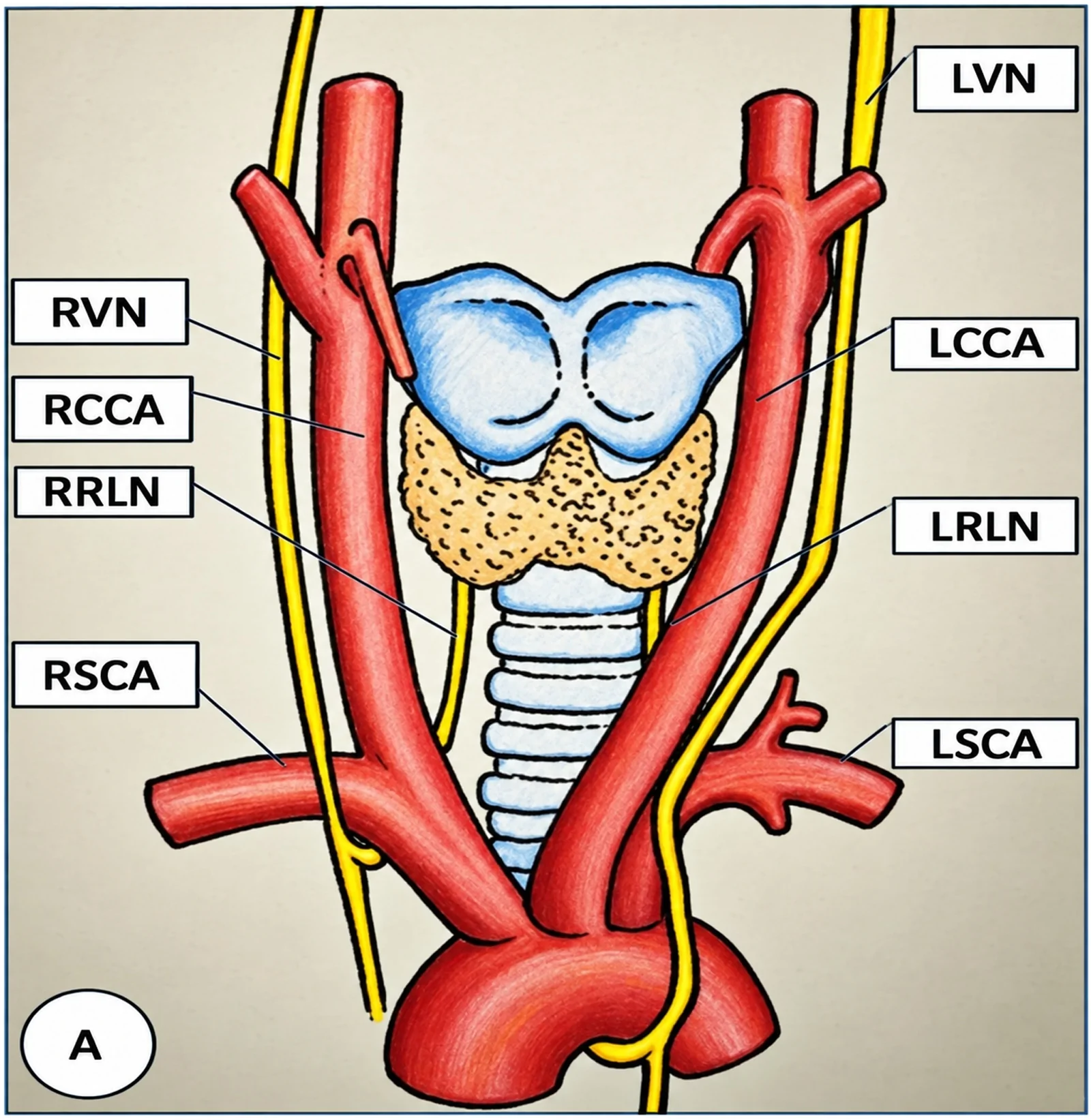
Anatomy of the recurrent laryngeal nerve. This schematic illustration demonstrates the distinct paths of the right and left recurrent laryngeal nerves, highlighting their relationship with the subclavian artery and the aortic arch.^36^ LCCA, Left Common Carotid Artery; LRLN, Left Recurrent Laryngeal Nerve; LSCA, Left Subclavian Artery; LVN, Left Vagus Nerve; RCCA, Right Common Carotid Artery; RRLN, Right Recurrent Laryngeal Nerve; RSCA, Right Subclavian Artery; RVN, Right Vagus Nerve.

In addition to the RLN, the vagus nerve has the Superior Laryngeal Nerve (SLN) as a branch in the neck. After descending toward the larynx, the SLN divides into the Internal Laryngeal Nerve (ILN) and External Laryngeal Nerve (ELN). The cricothyroid muscles, not innervated by the RLN, receives innervation from ELN, and adducts the vocal cords.^28^ The contraction of the cricothyroid muscles tilts the thyroid cartilage against the cricoid cartilage, and this motion increases the length and tension of the vocal folds, making a high-pitched voice. The other branch of the SLN, the ILN, provides sensory innervation to the laryngeal mucosa.^29^

According to Miyauchi and co-workers (2016), one of the major concerns in thyroid surgery is the possible change in the patient’s voice due to dysfunction of the laryngeal muscles.^30^ In 2018, 53.990 new cases of thyroid cancer are expected to be diagnosed in the United States.^31^ Henry and collaborators (2017) claim there are many exceptions to the normal innervation of the laryngeal muscles, which can influence the interpretation of laryngoscopy results or voice changes after thyroid surgery by physicians and speech therapists. A neural anastomosis that provides additional motor innervation to muscles normally innervated by the injured nerve can contribute to an incorrect interpretation of injury during laryngoscopy or stroboscopy.^28^ All of that makes it clear how important the knowledge of laryngeal innervation is.

The risk of injury to the RLN is ever present during neck surgery. Near the inferior pole of the thyroid gland, the right RLN is intimately related to the inferior thyroid artery and its branches (Fig. 6A). The RLN may cross anterior or posterior to branches of the artery, or it may pass between them. Due to this close association, the inferior thyroid artery is connected at a certain lateral distance from the thyroid gland, where it is not close to the nerve. Although the danger of injuring the left RLN during surgery is not as great owing to its more vertical ascent from the superior mediastinum, the artery and nerve are also closely associated near the inferior pole of the thyroid gland (Fig. 6B).^24^ The doctor Audrey Erman and collaborators (2009) published a paper showing the disorders of cranial nerve X (vagus) and concluded that unilateral RLN injury can be associated with phonation (voice production) disturbs, temporary aphonia (absence of voice) and laryngeal spasm. All the signs are commonly the result of bruising in RLN during a cervical surgery or from the pressure of accumulated blood and serous exudate after the operation.^32^

**Fig. 6:**
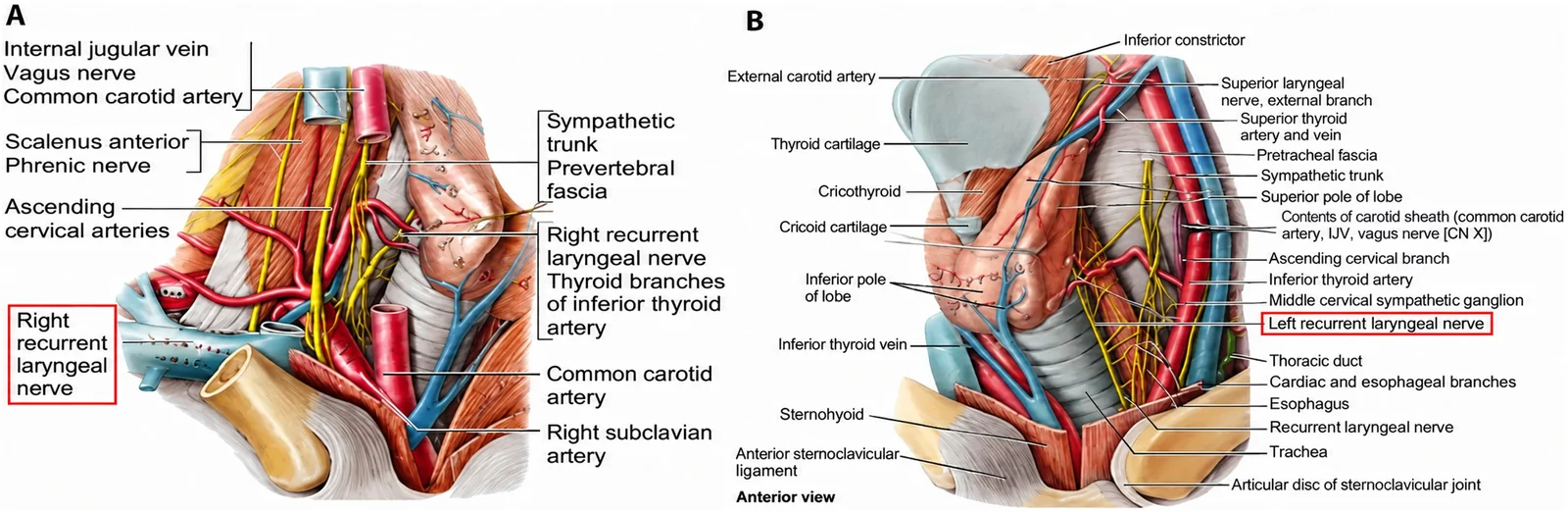
Anterior view of the inferior part of the neck. (A) The relationship between the right Recurrent Laryngeal Nerve (RLN) and the inferior thyroid artery. (B) The close association of the left RLN and the left inferior thyroid artery near the thyroid's inferior pole.^24^ RLN, Recurrent Laryngeal Nerve.

Both recurrent laryngeal nerves enter the larynx at the cricothyroid articulation through the fibers of the inferior constrictor muscles of the pharynx. During its ascent from the thorax to the larynx, numerous anatomic variations may be encountered.^33^ Several studies report that recurrent laryngeal nerves usually divide into two or more branches when approaching the inferior constrictor muscle before entering the larynx.^26^^,^^28^^,^^34^^,^^35^

Rustad (1954) showed that 43% of RLN divided into one or more branches on both sides. These branches may pass anteriorly or posteriorly to the cricothyroid articulation and then, coursing along the cricoarytenoid muscles innervates all the intrinsic laryngeal muscles, except of the cricothyroid. Nemiroff and Katz (1982) demonstrated that approximately 40% of RLN divide into two branches, and occasionally more, before entering in the larynx and this occurrence cannot be considered an anatomic rarity. Analyzing eighty adult cadavers (160-antimers), Tang and coworkers (2012) showed that 87.5% of the RLNs gave off multiple branches. There is a rare anatomic variation, (with a reported incidence of 0.3%‒0.8% on the right and 0.004% on the left side), when the nerve passes directly from the vagus in the neck towards the larynx and does not recur around right subclavian artery or aortic arch.^36^ This uncommon anatomic variation of the RLN that makes it highly susceptible to surgical injury is known as “nonrecurrent” inferior laryngeal nerve.^33^

## Discussion

This study confirms the overwhelming predominance of “Recurrent Laryngeal Nerve” (RLN) in scientific literature, with 1,828 MEDLINE/PubMed articles using this term in the title, compared with only 111 using “inferior laryngeal nerve”. This pattern holds across Embase, LILACS, SciELO, and Google Scholar. The Brazilian, international TA, and TNA editions officially recognize only “recurrent laryngeal nerve” (N. laryngeus recurrens, code TA A14.2.01.166).^37^ Critically, “inferior laryngeal” is currently reserved exclusively for vascular structures (vein and artery), not neural structures.^37^

Historically, “inferior laryngeal nerve” was common in 19th- and early 20th-century texts,^19^^,^^20^^,^^22^^,^^23^ particularly in European anatomical traditions, whereas “recurrent laryngeal nerve” gradually became preferred because it more accurately reflects the nerve’s retrograde course and offers greater anatomical clarity.^22^^,^^37^ Its adoption in modern literature and in the TA reflects this standardization. However, the persistence of “inferior laryngeal nerve” in contemporary literature likely reflects historical continuity and continued practical use. In some surgical and anatomical contexts, particularly in descriptions of branching near the larynx, the term is still used for the terminal portion of the recurrent laryngeal nerve rather than the entire nerve, which perpetuates terminological ambiguity. Older terminological traditions and its intuitive pairing with “superior laryngeal nerve” may also contribute to its residual use despite the broad consensus reflected in the TA, TNA, and most indexed publications.^38^

A temporal analysis of the search results further supports the consolidation of “recurrent laryngeal nerve” in contemporary scientific literature. In MEDLINE/PubMed, 439 of the 1,828 articles using this term in the title, corresponding to 24%, were published in the last five years, indicating sustained and active adoption. By contrast, only 10 of the 111 articles using “inferior laryngeal nerve”, or 9%, appeared during the same period. Similar patterns were observed in Embase, with 500 of 2,119 records versus 13 of 126, and in Google Scholar, with 770 of 3,420 records versus 22 of 214 since 2020. These findings show that, although “recurrent laryngeal nerve” clearly predominates and continues to expand in recent publications, “inferior laryngeal nerve” retains a small but persistent presence in the literature. This residual use reinforces the need for continued terminological standardization in research, teaching, and editorial practice.

Terminological precision is essential in both clinical practice and medical education, especially in cervical surgery (e.g., thyroidectomy). Ambiguous terminology hinders anatomical understanding, compromises surgical safety, and risks diagnostic or operative errors. RLN injury results in significant sequelae, including vocal changes and aphonia.^39^ Standardized nomenclature facilitates communication across medical specialties (surgery, otolaryngology, speech-language pathology) and optimizes laryngeal dysfunction evaluation.^2^ Conversely, inconsistency weakens didactic materials, clinical documentation, and, in research, diminishes the accuracy of literature searches and the reliability of systematic reviews.^12^ Terminological standardization is fundamental to surgical safety, international scientific integration, and educational uniformity.^12^

A precise understanding of the RLN's applied anatomy is also indispensable. The RLN, a vagal branch, ascends in the tracheoesophageal groove, near the thyroid’s inferior poles.^40^ It courses close to the inferior thyroid arteries.^40^ Due to high individual variability, Berry’s ligament is unreliable as a landmark; direct visualization is recommended during dissection.^41^ The left RLN follows a longer intrathoracic course around the aortic arch, while the right RLN loops around the subclavian artery.^40^ This difference (mean lengths: 9.4 cm left vs. 5 cm right) influences susceptibility to injury.^40^ Functionally, the RLN innervates all intrinsic laryngeal muscles except the cricothyroid, and provides sensory input to the infraglottic mucosa.^26^ Clinical RLN injury manifests as dysphonia and vocal fold paralysis.^42^ Left-sided vocal fold palsy is more often linked to thoracic causes because of the course of the left recurrent laryngeal nerve, supporting imaging of the mediastinal pathway.^43^ Preservation of the Recurrent Laryngeal Nerve (RLN) is critical in cervical surgeries. Transient paralysis rates range from 5% to 8%, while permanent paralysis occurs in 0.3%–3% of cases, with higher rates observed in reoperations, malignant disease, and extended thyroidectomies.^29^^,^^44^ Preventive strategies include direct visualization and Intraoperative Neurophysiological Monitoring (IONM).^45^ Anatomical variability is common, notably the aberrant right subclavian artery, which is associated with a non-recurrent laryngeal nerve in 86.7% of cases and profoundly alters the nerve's trajectory.^46^

Authors, reviewers, educators, and healthcare professionals are advised to consistently employ the term recurrent laryngeal nerve in accordance with current international standards.^12^^,^^37^ Scientific journals and institutions are urged to adopt official nomenclature in editorial policies and curricula. Routine consultation of the TA (www.unifr.ch/ifaa) should be encouraged. Study limitations include reliance on specific data sources and a primarily quantitative, descriptive methodology, without in-depth analysis of contextual usage across specialties or regions. Additionally, restricting the search to terms appearing only in article titles may be considered a study limitation, as this approach may have underestimated the overall frequency of both terms in the broader literature. However, this strategy provided a more conservative and methodologically consistent estimate of deliberate terminology choice, because searches of abstracts or full texts could capture incidental, comparative, or historical mentions that do not represent the primary designation adopted in the study.

## Conclusion

The adoption of standardized anatomical terminology is fundamental for effective scientific and clinical communication. Our findings support the recognition of “recurrent laryngeal nerve” as the most appropriate and recommended designation. Consistent use of this terminology strengthens anatomical education and improves the reliability and quality of scientific research.

Furthermore, a comprehensive understanding of the anatomy of the recurrent laryngeal nerve is indispensable for minimizing iatrogenic injury, particularly during neck surgery.

## ORCID ID

Jaiurte Gomes Martins da Silva: 0000-0002-6310-1023

Eduarda Correia Moretti: 0000-0002-3994-8689

Philippe Oliveira Lima: 0009-0008-1365-419X

Darlisson Rodrigues Duarte: 0009-0009-4297-3269

Abrahao Verçosa Amorim Filho: 0009-0004-4065-5471

Anderson Fernandes Silva: 0009-0001-6024-3843

Glícia Maria de Oliveira: 0000-0002-4415-5931

Jodonai Barbosa da Silva: 0000-0001-7432-8699

Ivson Bezerra da Silva: 0000-0002-0095-9671

Mariana de Carvalho Leal Gouveia: 0000-0003-3358-2791

## Declaration of Generative AI and AI-assisted technologies in the writing process

During the preparation of this work the authors used ChatGPT and Gemini in order to improve readability and language. After using these tools/services, the authors reviewed and edited the content as needed and takes full responsibility for the content of the publication.

## Funding

The authors received no financial support for the research, authorship, and/or publication of this article.

## Data availability statement

No new data were created or analyzed in this study.

## Declaration of competing interest

The authors declare that the research was conducted in the absence of any commercial or financial relationships that could be construed as a potential conflict of interest.
